# Definitive Airway Management of Patients with a King Laryngeal Tube^™^ in Place in the COVID-19 Pandemic

**DOI:** 10.5811/westjem.2020.4.47462

**Published:** 2020-04-24

**Authors:** Benjamin J. Sandefur, Brian E. Driver, Calvin A. Brown, Robert F. Reardon

**Affiliations:** * Mayo Clinic College of Medicine and Science, Department of Emergency Medicine, Rochester, Minnesota; † Hennepin County Medical Center, Department of Emergency Medicine, Minneapolis, Minnesota; ‡ Brigham and Women’s Hospital and Harvard Medical School, Department of Emergency Medicine, Boston, Massachusetts

To the Editor,

The COVID-19 pandemic has generated enhanced focus on the safety of healthcare providers and efforts to mitigate the risks of viral transmission.^1^ Reports of previous viral epidemics have described substantially increased risk to providers performing laryngoscopy and tracheal intubation in patients infected with the virus.^2^,^3^ Additionally, bronchoscopy and other endoscopic airway procedures are considered high-risk, aerosol-generating procedures.^1^

The King LT(S)-D laryngeal tube (King Systems, Noblesville, IN), abbreviated hereafter as the King LT, is a new-generation extraglottic device (Figure 1) used as a primary or backup airway device by many emergency medical systems systems. This device has been demonstrated to have advantageous attributes as compared to other extraglottic airway devices, with favorable safety outcomes and high rates of successful insertion.^4^–^7^ However, the King LT is not a definitive airway device and is not intended for long-term use. Additionally, the King LT has been associated with post-insertion airway edema, which, in addition to risk factors inherent to the patient, may further impede subsequent laryngoscopy attempts.^8^,^9^ Early exchange of a King-LT for an endotracheal tube is important in reducing this risk. An endoscopic Seldinger-style technique for tracheal tube placement using an Arndt airway exchange catheter (Cook Medical, Bloomington, IN) has been described.^9^ However, this technique may increase generation of aerosols containing highly infectious viral particles. Additionally, many emergency physicians may be unfamiliar with this approach or lack the necessary endoscopic equipment. Given the current COVID-19 pandemic, emergency physicians need to have a straightforward, safe approach for definitive airway management in patients with a King-LT using airway equipment commonly found in the emergency department (ED).

In 2016, Dodd and colleagues introduced a novel, nonsurgical approach to facilitate definitive airway management in ED patients with a King LT in place.^10^ The authors described use of a standard-geometry video laryngoscope and bougie to intubate the trachea with the King LT device remaining in situ. A bougie is used, instead of initial intubation with a tracheal tube, given its smaller diameter and the inherent space limitation that the King LT imposes within the pharynx where the devices are manipulated. Furthermore, the on-screen visualized supraglottic region might be obscured as the larger endotracheal tube passage is attempted, while use of a bougie results in less obstruction of the visualized field. The authors reported a 99.8% success rate with this nonsurgical and non-endoscopic technique, and noted that in rare cases of failed intubation, the King LT remains in a functional position allowing for balloon reinflation and resumption of ventilation. A subsequent, proof-of-concept cadaveric study demonstrated similar (100%) first-pass success, although the authors acknowledged the potential for overestimation given the small sample size.^11^ This concept was demonstrated in real-world clinical practice in an observational study of 647 patients arriving to the ED with a prehospital-placed King LT.^12^ In this study 112 of 647 patients underwent intubation with the King LT left in place, with the balloons deflated, with first-attempt success in 102 (91%). Of the 10 patients with first-attempt failure, eight patients were intubated with the same technique on the second attempt; the remaining two were intubated with bougie facilitation after removing the King LT.

We believe that the King LT exchange method described by Dodd and colleagues represents a safe and simple approach that can be readily performed by clinicians who are skilled at video laryngoscopy. Further, we believe that this method, used in combination with administration of a high-dose, paralytic medication to mitigate spontaneous patient respiration and cough during the procedure, represents the safest method for both patients and care providers to exchange a King LT for a cuffed tracheal tube in a patient with known or suspected COVID-19. Lastly, the airway equipment required is readily available to most emergency providers, critical care providers, and anesthesiologists. We describe this procedure, with updates accounting for risks inherent to the COVID-19 pandemic, in Figures 2 and 3.

## Figures and Tables

**Figure 1:**
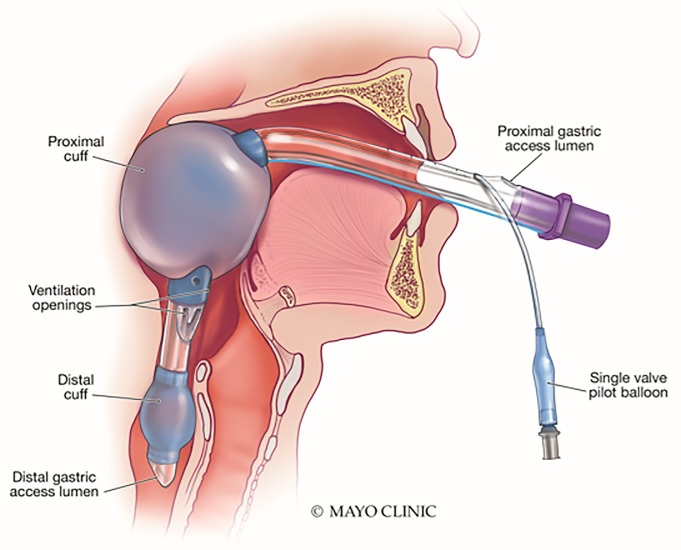
King LT(S)-D^™^ laryngeal tube (King Systems; Noblesville, IN, USA). From Subramanian A, Garcia-Marcinkiewicz A, Brown D, et al. Definitive airway management of patients presenting with a pre-hospital inserted King LT(S)-D^™^ laryngeal tube airway: a historical cohort study. *Can J Anesth.* 2016;63(3):275–82. Printed with permission of Mayo Foundation for Medical Education and Research, all rights reserved.

**Figure 2:**
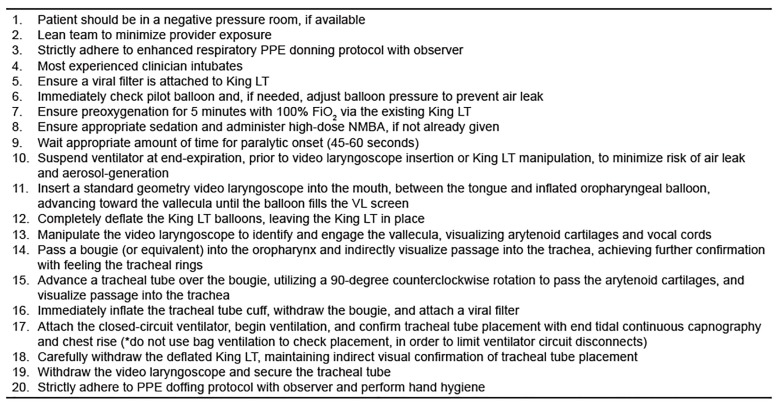
Safe Approach to King Exchange in Patients with COVID-19. *If the procedure fails at any point, the King LT can be reinflated and used for oxygenation and ventilation. *PPE*, personal protective equipment; *LT*, laryngeal tube; *FiO_2_*, fraction of inspired oxygen; *NMBA*, neuromuscular blocking agent; *VL*, videolaryngoscopy.

**Figure 3:**
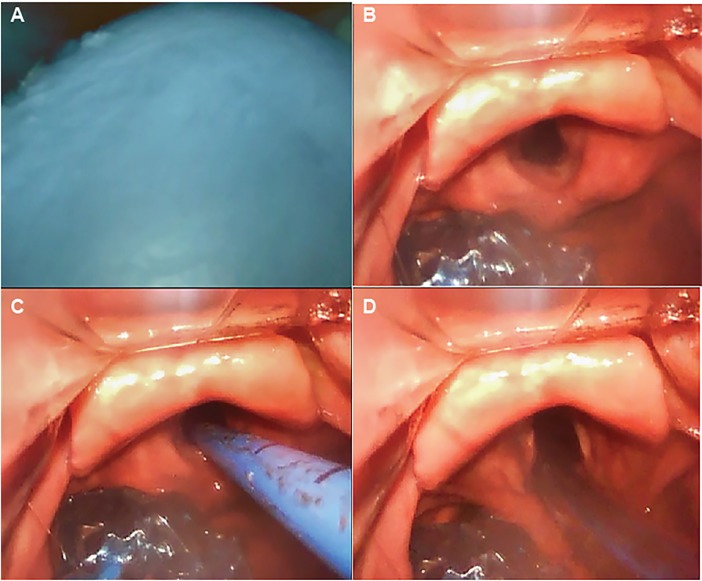
Steps for tracheal intubation with the King LT in situ. A. After suspending ventilator at end-expiration, the clinician advances the video laryngoscope into the oropharynx, along the superior surface of the tongue (top) and anterior to the King LT (bottom). The oropharyngeal balloon can be visualized filling the screen. B. The King LT balloons are deflated, and blade is advanced into the vallecula, with arytenoid cartilages and vocal cords visualized on the screen. C. The clinician passes a bougie into the trachea, with visual confirmation and confirmation from feel of tracheal rings D. The tracheal tube is advanced over the bougie, utilizing a 90-degree counterclockwise rotation to avoid encountering the arytenoid cartilages. *After confirmation of tracheal intubation, the King LT is removed. If the procedure fails at any point, the King LT can be reinflated and used for oxygenation and ventilation. Figure 3, Image A courtesy of Robert F. Reardon, MD and Figure 3; Images B-D courtesy of Benjamin J. Sandefur, MD.

## References

[1] World Health Organization Clinical management of severe acute respiratory infection (SARI) when COVID-19 disease is suspected. Interim Guidance.

[2] Raboud J, Shigayeva A, McGeer A (2010). Risk Factors for SARS Transmission from Patients Requiring Intubation: A Multicentre Investigation in Toronto, Canada. PLoS One.

[3] Tran K, Cimon K, Severn M (2012). Aerosol Generating Procedures and Risk of Transmission of Acute Respiratory Infections to Healthcare Workers: A Systematic Review. PLoS One.

[4] Russi CS, Miller L, Hartley MJ (2008). A comparison of the King-LT to endotracheal intubation and Combitube in a simulated difficult airway. Prehosp Emerg Care.

[5] Tumpach EA, Lutes M, Ford D, Lerner EB (2009). The King LT versus the Combitube: flight crew performance and preference. Prehosp Emerg Care.

[6] Schalk R, Byhahn C, Fausel F (2010). Out-of-hospital airway management by paramedics and emergency physicians using laryngeal tubes. Resuscitation.

[7] Russi CS, Hartley MJ, Buresh CT (2008). A pilot study of the King LT supralaryngeal airway use in a rural Iowa EMS system. Int J Emerg Med.

[8] Gaither JB, Matheson J, Eberhardt A, Colwell CB (2010). Tongue engorgement associated with prolonged use of the King-LT laryngeal tube device. Ann Emerg Med.

[9] Subramanian A, Garcia-Marcinkiewicz A, Brown D (2016). Definitive airway management of patients presenting with a pre-hospital inserted King LT(S)-DTM laryngeal tube airway: a historical cohort study. Can J Anesth.

[10] Dodd K, Klein L, Kornas R (2016). Definitive airway man-agement in emergency department patients with a King laryngeal tube in place: a simple and safe approach. Can J Anesth.

[11] Dodd K, Kornas R, Prekker M (2017). Endotracheal intubation with the King laryngeal tube in situ using video laryngoscopy and a bougie: a retrospective case series and cadaveric crossover study. J Emerg Med.

[12] Driver BE, Scharber SK, Horton GB, Braude DA, Simpson NS, Reardon RF (2019). Emergency Department Management of Out-of-Hospital Laryngeal Tubes. Ann Emerg Med.

